# Detection of dengue-4 virus in pune, western india after an absence of 30 years - its association with two severe cases

**DOI:** 10.1186/1743-422X-8-46

**Published:** 2011-02-01

**Authors:** Dayaraj Cecilia, Mahadeo B Kakade, Asha B Bhagat, Joyprashant Vallentyne, Anand Singh, Jayashri A Patil, Shankar M Todkar, Sunitha B Varghese, Paresh S Shah

**Affiliations:** 1 National Institute of Virology, 20-A, Dr Ambedkar Road, Pune-411001, Maharashtra, India; 2 Todkar Hospital, 8/1 Mangalwar Peth, Pune-411014, Maharashtra, India; 3 Niramaya Hospital, Chinchwad, Pune-411019, Maharashtra, India

## Abstract

**Background:**

Difference in severity of dengue outbreaks has been related to virus serotype, genotype and clades within genotypes. Till the 1980 s, India and Sri Lanka reported low number of dengue hemorrhagic fever (DHF) cases despite circulation of all four serotypes of dengue virus (DENV). Since the 1990 s the occurrence of DHF has increased. The increase has been attributed to changes in virus lineage especially with regard to DENV-2 and DENV-3. DENV-1 has been associated with dengue fever (DF) outbreaks and DENV-4 reports have been rare. The emergence of DENV-4 was reported recently in 2003 in Delhi and in 2007 in Hyderabad. The last report of DENV-4 from Maharashtra was in 1975 from Amalner.

**Results:**

We report on the detection of DENV-4 in Pune, Maharashtra after an absence of almost 30 years. Two cases were detected in 2009-10, serotyped by multiplex reverse transcriptase polymerase chain reaction (RT-PCR). Both the cases were recorded as severe dengue (Category 3) requiring intensive care unit (ICU) level of treatment. Depending on the hemagglutination inhibiting (HI) antibody titres the 2009 case was characterized as a primary infection and the 2010 case as a secondary infection. Both the cases presented plasma leakage and neither showed any kind of haemorrhage. The 2009 case survived while the 2010 case was fatal. An isolate was obtained from the 2009 case. Based on envelope (E) gene sequence analysis, the virus belonged to genotype I of DENV-4, and clustered with isolates from India and Sri Lanka and was distant from the isolates from Thailand. The nucleotide and amino acid diversity of the E gene of the Indian isolates increased from 1996 to 2007 to 2009 in context of the E gene sequences of other isolates belonging to genotype I.

**Conclusion:**

The increasing diversity in the circulating DENV-4 calls for close monitoring of the DENV-4 serotype.

## Approach

The National Institute of Virology is the WHO Collaborating Centre For Arbovirus And Hemorrhagic Fever Reference And Research. We work in close collaboration with clinicians in providing dengue diagnosis. Samples from suspected dengue cases are tested for dengue specific IgM, using NIV MAC-ELISA kit [1], viral RNA using dengue-specific real time RT-PCR [2] and serotyped by multiplex nested RT-PCR test [3]. As a gold standard, virus isolation is attempted by infecting C6/36 cells (*Aedes albopictus *mosquito cell line) with patient sera. The infected cells are examined for the presence of virus by immunofluorescence assay (IFA) and RT-PCR. Sequencing of viral RNA is carried out using big dye terminator kit (Applied Biosystems, Foster city, CA, USA). The infection is characterized as primary or secondary based on the HI antibody response.

## Findings

Our studies on Dengue in Pune from 2002 to 2008 revealed that DENV-1, 2 and 3 were co-circulating in Pune (unpublished data). From May 2009 to September 2010, 56 cases could be serotyped by the multiplex RT-PCR test. Thirteen cases of DENV-1, 21 of DENV-2, 20 of DENV-3 and two of DENV-4 were detected. The serotype was confirmed by sequencing the amplicon. The first DENV-4 case, Case 1, occurred at the end of the seasonal outbreak period in November 2009. The second case, Case 2 was in the early phase of the season in June 2010. The two cases were hospitalised patients and underwent standard daily clinical evaluation and physical examinations. The case history forms of the patients were filled from the time of admission. Neither of the patients had any recorded history of dengue infection in the past. Both cases presented severe manifestations and required ICU level of care. They could thus be considered as Category 3 (patients requiring bed rest, intensive care-unit level observation protocol) according to the new WHO classification system, which depends on the intervention protocol [4]. Both the cases presented the common symptoms of dengue and symptoms indicative of plasma leakage (Table 1). Case 1 recovered while Case 2 died. Both cases had thrombocytopenia, however the counts normalized by day 5 (post hospitalisation) in Case 1 while it continued to decline in Case 2. Both cases had no symptoms of haemorrhage. There was mild ascites in Case 1, who survived and pleural effusion in Case 2, who died. Respiratory distress has been reported in death cases of dengue [5,6]. Case 2 had severe liver damage as indicated by the dramatic increase in the ALT/AST levels (>1000 IU/L). Liver damage is one of the major symptoms reported for DHF/DSS cases in India [7].

**Table 1:** Clinical profile of patients infected with DENV-4

	Case1 - 2009 (0952326)	Case2 - 2010 (1014847)
Age/Sex	27/M	54/F

IgM	+	-

Fever	+	+

Headache	+	+

Body pain	+	+

Rash	-	+

Nausea/vomiting	+	+

Chills	-	+

Pulse (/min)^@^	92^1^, 88^2^	80-90^1 ^, 60-70^2^

Blood pressure^@^	100/90^1^, 120/80^2^	130/80^1^, 90/60^2^

Platelets/cumm*	24000, 12000, 17000, 42000, 92000	124000, 100000, 34000, 11000

Hemoglobin	15.0	16.4

WBC Total/cumm*	1900,5500, 6300	2300,3500,2900,4700

Hepatomegaly	+	+

Splenomegaly	+	-

Pleural effusion	-	+

Ascites	Mild +	-

Serum albumin (g%)	5.7	2.8

Serum ALT (normal up to 40 U/L)	145 IU/L	1030 IU/L

Serum AST (normal up to 30 U/L)	257 IU/L	2500 IU/L

Serum bilirubin (normal <1 mg%)	0.62 mg%	1.2 mg%

* Platelet counts were taken every day from 1^st ^day of hospitalisation

^@1 ^- Day of admission.

^@2 ^- Day of discharge/death.

DENV-specific IgM antibodies were assessed in serum samples collected on 5^th ^day post onset of illness for Case 1 and 4^th ^day post onset of illness for Case 2. Case 1 was positive for IgM while Case 2 was negative. The titre of HI antibodies in the serum was determined to define whether the individuals had suffered primary or secondary infections. HI antibody titre of >1:2560 in the acute phase of infection is considered confirmatory of secondary infection [8]. Case 1 had very low levels of HI antibodies (1:40 to 1:80) indicating a primary infection. Case 2 had HI titre of 1:2560 indicating a secondary infection. The serum samples were also tested in the dengue IgG capture ELISA (Panbio Ltd.) and showed the presence of 31 units of IgG in Case 1 and 78 units in Case 2.

**Figure 1:**
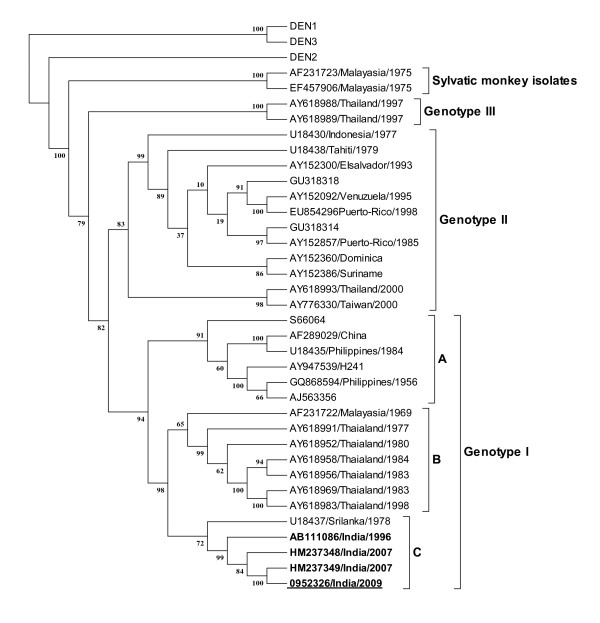
**Phylogenetic analysis of Dengue 4 based on the Envelope (E) gene sequence**. The ML tree was constructed using the PhyML 3.0 [9] software. Bootstrap values for 1000 replicates are indicated on each branch. The scale at the bottom indicates the number of nucleotide substitutions per site. The Indian strains are indicated in bold letters, HM indicates Hyderabad and the isolate sequenced in the present study is underlined.

DENV-4 as the aetiological agent was confirmed by multiplex RT-PCR and sequencing of the amplicon. Sequence analysis of the 500 bp fragment, which represented the core-prM region, revealed that the 2009 and 2010 viruses had high similarity with each other (>99%). An isolate was obtained from Case 1 by infecting C6/36 cells with the patient serum, no isolate could be obtained from the serum of Case 2. The envelope (E) gene was sequenced from the virus obtained after a single passage (HQ600557) and analysed in context with other sequences of DENV-4, present in the GenBank using Maximum Likelihood method, PhyML_3.0 [9]. To date, four genotypes of DENV-4 virus have been described consisting of viruses from Southeast Asia (genotype I), Southeast Asia and the Americas (genotype II), Thailand (genotype III) and Malaysia (Sylvatic) [10]. The 2009 isolate belonged to genotype I or the South East Asian genotype (Figure 1). Within the genotype there were three major clusters representing viruses from A) China and Philippines, B) Malaysia and Thailand and C), India and Sri Lanka suggesting the presence of clades within the genotype. The DENV-4 isolates even within the Indian cluster showed high diversity in the E gene. The Pune 2009 isolate (0952326/India/2009) showed a diversity of >4% as compared to the other Indian isolates of cluster A and 7% as compared to a Thailand isolate from cluster B. The amino-acid diversity ranged between 1.6% to 3.1%. The diversity in nucleotide as well as amino acid sequence of the E gene of Indian isolates increased from 1996 [GenBank: AB111086] to 2007 [GenBank: HM237348] to 2009 (0952326/India/2009) as shown by the arrows in Additional file 1: Table S1, when compared to the other isolates from the region i.e Sri Lanka, Malaysia and Thailand. The nucleotide diversity observed between the DENV-4 isolates was much higher than that reported for DENV-2 isolates within a particular genotype [11]. To strengthen the data and carry out in depth analysis, isolates from the other centres of India reporting the circulation of DENV-4 need to be sequenced.

## Conclusion

The high degree of diversity in the envelope gene observed for the DENV-4 viruses circulating on the subcontinent indicates that the serotype is evolving and that close monitoring of the serotype is needed.

## Competing interests

The authors declare that they have no competing interests.

## Authors' contributions

DC designed and coordinated the study and wrote the manuscript. MK carried out real time RT-PCR and sequencing. AB was involved in virus isolation. JV carried out the serotyping and alignment analysis. AS carried out the serological tests. JP carried out the ML analysis. ST and SV are the clinicians involved and provided the clinical data. PS coordinated with the hospitals for diagnosis and sampling.

All authors read and approved the final manuscript

## Supplementary Material

Additional file 1 **Table S1: Nucleotide/Amino acid diversity of E gene of DENV-4 isolates of Genotype I**. The lower diagonal half presents the nucleotide diversity while the upper half represents the amino acid diversity between the DENV-4 isolates selected from genotype I. The arrows indicate the increasing values of diversity from 1996 to 2007 to 2009. Click here for file
