# Supplementary material for: Detection of dengue-4 virus in pune, western india after an absence of 30 years - its association with two severe cases
Source: Virol J. 2011 Feb 1;8:46. doi: 10.1186/1743-422X-8-46 (PMC3041772; doi:10.1186/1743-422X-8-46)
Supplement: Additional file 1 — Table S1: Nucleotide/Amino acid diversity of E gene of DENV-4 isolates of Genotype I. The lower diagonal half presents the nucleotide diversity while the upper half represents the amino acid diversity between the DENV-4 isolates selected from genotype I. The arrows indicate the increasing values of diversity from 1996 to 2007 to 2009. [file 1743-422X-8-46-S1.DOC]

|  | 0952326/  Pune,  India ‘09 | [HM237348] Hyderabad India ‘07 | [AB111086] India ‘96 | [U18437]  Sri Lanka ‘78 | [AF231722] Malaysia ‘69 | [AY618983]  Thailand ‘98 |
| --- | --- | --- | --- | --- | --- | --- |
| 0952326/  Pune,  India ‘09 |  | 0.027 | 0.016 | 0.029 | 0.020 | 0.031 |
| [HM237348] Hyderabad India ‘07 | 0.044 |  | 0.010 | 0.020 | 0.010 | 0.014 |
| [AB111086] India ‘96 | 0.040 | 0.036 |  | 0.014 | 0.004 | 0.014 |
| [U18437]  SriLanka ‘78 | 0.046 | 0.037 | 0.033 |  | 0.014 | 0.025 |
| [AF231722] Malaysia ‘69 | 0.042 | 0.032 | 0.028 | 0.019 |  | 0.042 |
| [AY618983]  Thailand ‘98 | 0.073 | 0.056 | 0.056 | 0.052 | 0.014 |  |
